# Supplementary material for: Association between oral microbiome and seven types of cancers in East Asian population: a two-sample Mendelian randomization analysis
Source: Front Mol Biosci. 2023 Nov 21;10:1327893. doi: 10.3389/fmolb.2023.1327893 (PMC10702768; doi:10.3389/fmolb.2023.1327893)
Supplement: Supplementary file 1 [file DataSheet1.ZIP › Supplementary Materials/MR plots for tongue/tongue═╝/Colorectal cancer/pheno.3333_to_colorectal cancer_forest.pdf]

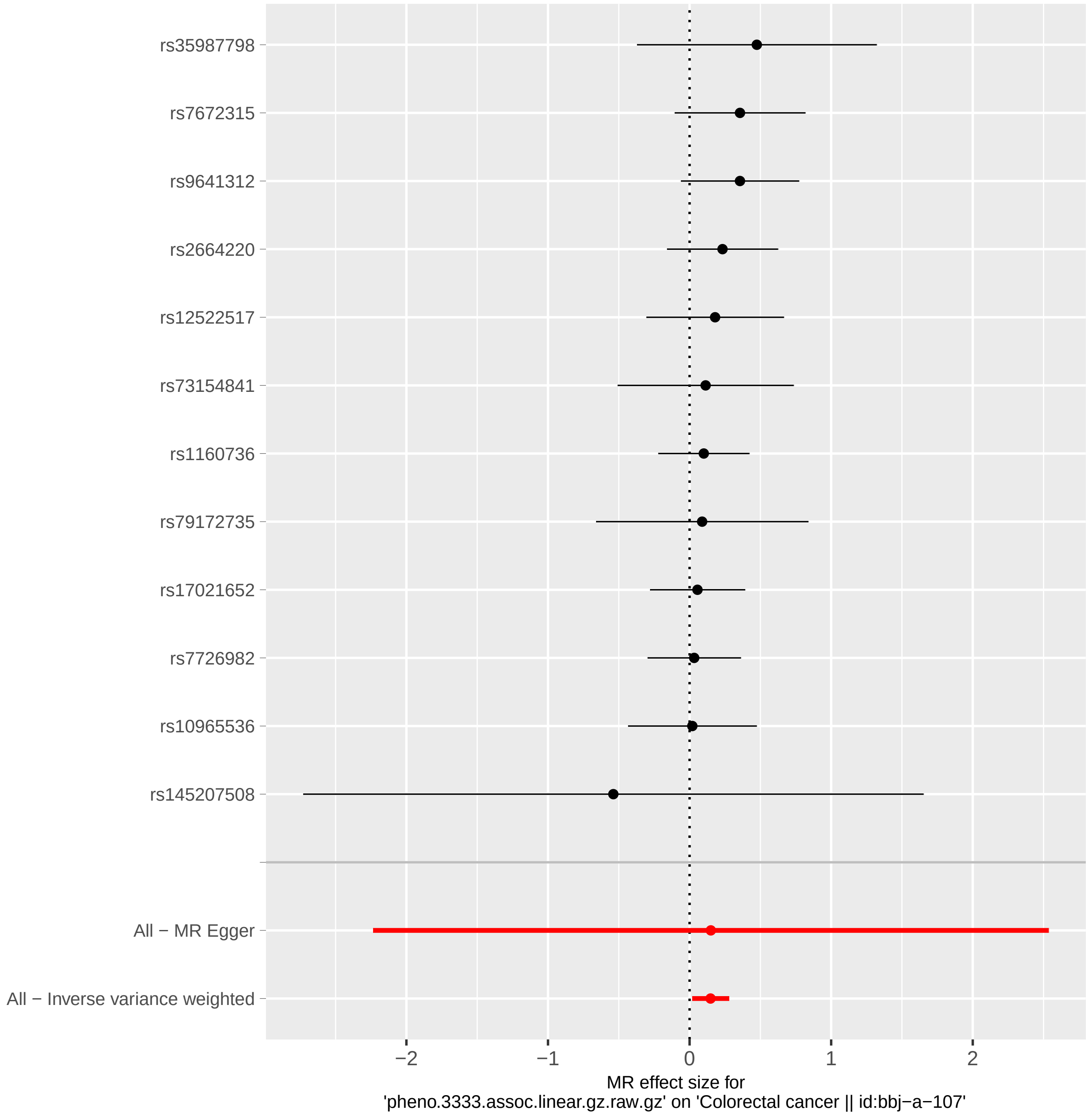
rs35987798
rs7672315
rs9641312
rs2664220
rs12522517
rs73154841
rs1160736
rs79172735
rs17021652
rs7726982
rs10965536
rs145207508
All − MR Egger
All − Inverse variance weighted
−2
−1
0
1
2
MR effect size for
'pheno.3333.assoc.linear.gz.raw.gz' on 'Colorectal cancer || id:bbj−a−107'
